# Survey of Synanthropic Spiders in Ireland Reveals Expansion and Dominance of the Invasive Noble False Widow *Steatoda nobilis* in Urban Habitats (Araneae: Theridiidae)

**DOI:** 10.1002/ece3.73193

**Published:** 2026-03-10

**Authors:** Brandon L. Collier, Dayle Leonard, Keith Lyons, John P. Dunbar, Colin Lawton, Michel M. Dugon

**Affiliations:** ^1^ Venom Systems & Proteomics Lab, School of Natural Sciences, Martin Ryan Institute University of Galway Galway Ireland; ^2^ Midlands Bug and Reptile Zoo Longford Ireland; ^3^ Animal Ecology & Conservation Unit, School of Natural Sciences, Martin Ryan Institute University of Galway Galway Ireland

**Keywords:** anthropogenic, false widow, invasive species, spider ecology, synanthropic, urban spiders

## Abstract

Rapid urbanisation has led several spider species to adapt to synanthropic microhabitats and establish large populations outside of their native ranges. In Ireland, the establishment and widespread distribution of the Noble false widow spider *Steatoda nobilis* (Araneae: Theridiidae) (Thorell, 1875) has raised questions about its impact on native spider populations across the country. Through an extensive field survey of six urban centres over an 11‐month period, we sought to establish population demographics for *S. nobilis* and other synanthropic spiders in Ireland for the first time. We surveyed fence microhabitats for both spider abundance and diversity to determine the influence of variables such as prey availability and climate. Of the 20 identifiable species observed, *S. nobilis* and the missing sector orb weaver *Zygiella x‐notata* (Clerck, 1757) typically made up more than 80% of the spider abundance regardless of location surveyed, including two new Irish counties where *S. nobilis* had previously not been recorded but is now well established (Co. Mayo and Co. Sligo). Our results also indicated that the diversity of synanthropic spiders is significantly affected by seasonality and prey availability, but largely unaffected by daily weather conditions.

## Graphical abstract

Rapid urbanisation has facilitated the establishment of large populations of the Noble false widow spider outside of its native range through the use of synanthropic microhabitats. In Ireland, we survey these favourable microhabitats for spider diversity for the first time and find that the Noble false widow now typically makes up more than 40% of the assemblage.

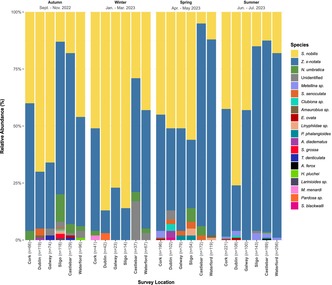

## 1 Introduction

In the Anthropocene, some spiders of the family Theridiidae (Araneae) have rapidly dispersed from their native ranges to become cosmopolitan, and occasionally invasive, species. Members of the subfamily Latrodectinae have been at the forefront of this trend; the brown widow *Latrodectus geometricus* (Koch, 1841) and the redback spider *Latrodectus hasseltii* (Thorell, 1870) have spread from their respective African and Australian ranges, and have established populations in both the Northern and Southern hemispheres (Garb et al. 2004; Taucare‐Ríos, Bizama, and Bustamante 2016; Vetter et al. 2012; Vink et al. 2011; Wahlberg et al. 2023). Likewise, the Noble false widow *Steatoda nobilis* (Thorell 1875) was first observed outside of its hypothesised native range of the Macaronesian archipelago (Bristowe 1929; Pickard‐Cambridge 1907; Thorell 1875) over 140 years ago, when a specimen was found in Torquay, England (Pickard‐Cambridge 1879). After remaining confined to a handful of port cities in Great Britain for over a century, the species started to spread globally at a very fast rate from the mid‐to‐late 1990's onwards. *S. nobilis* has established dense populations in North America (i.e., California) (Vetter et al. 2015; Vetter and Rust 2012), South America (i.e., Chile and Ecuador) (Bauer et al. 2019; Faúndez et al. 2018; Faúndez and Téllez 2016; Taucare‐Ríos, Mardones, and Zúñiga‐Reinoso 2016), North Africa (i.e., Morocco) (Denis 1962), West Asia (i.e., Turkey and Iran) (Zamani et al. 2015), and across most western European nations from Ireland to Greece (Emerit and Ledoux 2013; Kulczycki et al. 2012; Reiser 2013; Snazell and Jones 1993). More recently, *S. nobilis* has also established a growing population in New Zealand (Picknell and Trewick 2025). Although the exact causes for the global extension of its range are unknown, it is notable that the early radiation and recent rapid spread of *S. nobilis* match closely the increase in global trading and tourism routes (Bauer et al. 2019). Global trade and mass tourism have also been shown to vector other arthropod invasions (Bonnamour et al. 2021).

Of the cosmopolitan Theridiid species, two false widow species, *Steatoda grossa* (Koch, 1838) and *S. nobilis* , have established on the island of Ireland (Dugon et al. 2017; Nolan 1999). *S. nobilis* is a comparatively large member of the *Steatoda* genus; with the females (9.5 mm to 14 mm in length) being generally larger than the males (7 to 11 mm) (Dugon et al. 2017; Snazell and Jones 1993). *S. nobilis* have also adapted a very flexible hunting strategy, utilising webs with a three‐dimensional structure and designated threads for catching both flying and ground‐dwelling prey (Dugon et al. 2017, 2023; Dunbar, Ennis, et al. 2018; Dunbar et al. 2022). Unlike other Theridiid spider species, *S. nobilis* appears to exhibit a wide range of temperature and humidity tolerance, establishing in desertic, sub‐tropical, semi‐desertic, and cool, humid temperate climates (Bristowe 1929; Dugon et al. 2017; Nolan 1999; Pickard‐Cambridge 1879, 1907; Taucare‐Ríos et al. 2025; Thorell 1875). Because it typically forms large populations in urban environments, *S. nobilis* has likely been able to overcome some of the unique stressors associated with synanthropic environments (e.g., urban heat effects, habitat structures, pollution) (Willmott et al. 2025). Likely because in part of these competitive traits, *S. nobilis* has rapidly expanded its distribution and the density of its populations throughout Ireland since its establishment around the late 1990s (Dugon et al. 2017; Dunbar, Schulte, et al. 2018; Nolan 1999).

Several papers have previously called for research into the ecological impact of *S. nobilis* (e.g., Dugon et al. 2017; Kulczycki et al. 2012; Picknell and Trewick 2025). As the species continues to expand its range globally, there is also pressure to identify any possible effects that the establishment of *S. nobilis* may have on native species, including other spiders. Kulczycki et al. (2012) noted that Italian populations of orb‐weaver spiders from the genus *Zygiella* seemed to be “largely substituted by those of *S. nobilis* on gates, lamp posts, and along railings”, supporting the idea that *S. nobilis* may be capable of displacing native synanthropic spiders in novel ranges. To determine the potential implications of its presence on native Irish spiders, Rayner et al. (2022) explored how *S. nobilis* compared to several synanthropic spider species in terms of intraguild competition (i.e., the ability to kill members of a competing species to win resources). *S. nobilis* specimens were placed in a controlled setting with several common synanthropic spiders found in Ireland to monitor if predation between the species occurred. In 97.5% of interactions within the cohorts (*n* = 40), *S. nobilis* was able to disable and feed on the other competing spiders; the one exception being a single instance of *S. nobilis* being preyed upon by the lace‐web spider *Amaurobius similis* (Blackwall, 1861). In essence, Rayner et al. (2022) showed that *S. nobilis* can outcompete common synanthropic Irish species in a laboratory setting. However, the potential impacts of *S. nobilis* on synanthropic spider populations in situ has yet to be explored. Laboratory results do not always reflect how interspecific and intraspecific interactions are affected by abiotic and biotic factors, such as seasonal climate, daily weather conditions, and prey availability. Several synanthropic spider species are known to utilise urban refugia to mitigate the impact of environmental variables (Roberts et al. 2025), but the impact of such variables in areas with limited refugia has yet to be explored. In addition, examining the spiders in the field could provide vital insights into whether *S. nobilis* has a competitive, displacement‐causing (and therefore invasive) impact on native spiders.

Here, we used field survey techniques to characterise the relative abundance and biodiversity of Ireland's synanthropic spider populations for the first time. In doing so, we investigate the effect of *S. nobilis* on other spider species within favourable urban microhabitats across the Republic of Ireland. We conducted a series of surveys and experiments over an 11‐month period to (1) assess the current status of *S. nobilis* relative to other synanthropic spider species in Ireland, and (2) investigate how synanthropic spider populations in Ireland are affected by changes in seasonal climate, daily weather conditions (temperature, rainfall, wind speed), and prey availability. Finally, we use this data to explore the invasive status of *S. nobilis* in Ireland as well as its potential impact if introduced to highly endemic and protected insular habitats around the world.

## 2 Methods

### 2.1 Field Techniques

In urban areas, structures such as residential buildings, walls, graves, and street furniture have been shown to house many species of spiders regardless of their synanthropic status (Fossitt 2000). However, many of these built structures are not suitable locations for carrying out arthropod surveys. Stone walls, for example, are common fixtures across Ireland, but it is almost impossible to survey in detail because of the number of potential hides each metre of wall holds, making it difficult to account for every spider present in many instances. Metal property fences, however, provide a solid structure with enough shelter and open space to accommodate species that hunt flying and/or walking prey while presenting few visual impediments. Many Irish spider species have also been documented colonising and maintaining populations on fences (Christoph and Milan 2018; Dugon et al. 2017; Kish and Henkanaththegedara 2019; Kralj‐Fišer et al. 2014, 2017), so the present study focuses specifically on these microhabitats.

Dugon et al. (2017) established that there are at least 16 Irish counties with records of *S. nobilis* sightings. All specimens were observed in the direct vicinity of human settlements/structures, mostly within larger cities and towns, and the species seems to be absent from any remote, natural environment (e.g., woodlands, bogs, or grasslands). In the current study, six urban areas were chosen: Dublin City (Co. Dublin), Cork City (Co. Cork), Waterford City (Co. Waterford), Galway City (Co. Galway), Sligo Town (Co. Sligo), and Castlebar Town (Co. Mayo). The four locations with the largest human populations of the set (Dublin, Cork, Waterford, and Galway) were chosen as they have at least one published record of *S. nobilis* . Additionally, both Sligo Town and Castlebar were selected as potential control sites, as both towns are located furthest from the hypothesised introduction point for *S. nobilis* (i.e., the east coast), in counties with relatively low human population density. Additionally, the status of *S. nobilis* in these urban centres was unknown, with searches performed in 2016/2017 in both Co. Sligo and Co. Mayo yielding no *S. nobilis* specimens, although it was speculated that a more comprehensive survey could produce them (Dugon et al. 2017).

Google Earth Pro (Map of the Republic of Ireland 2022) was utilised to identify appropriate fences for the survey. Only public fences and those surrounding businesses, religious edifices, and large apartment complexes were deemed suitable for study. In larger cities, sites were also selected on the basis of their proximity to one another and their ability to represent different habitats within their respective locations. In Dublin City, for example, several sites in the western town of Lucan were chosen to represent the densely populated suburban areas of the city. Ten suitable fences were chosen for each of these urban centres (Table S1). This constituted a database from which the survey sites could be randomly selected without introducing further sampling bias. Five fences for each urban centre from this database were randomly selected using RANDOM.ORG (Haahr 2023) to be surveyed for biodiversity.

### 2.2 Synanthropic Spider Biodiversity Survey

**FIGURE 1:**
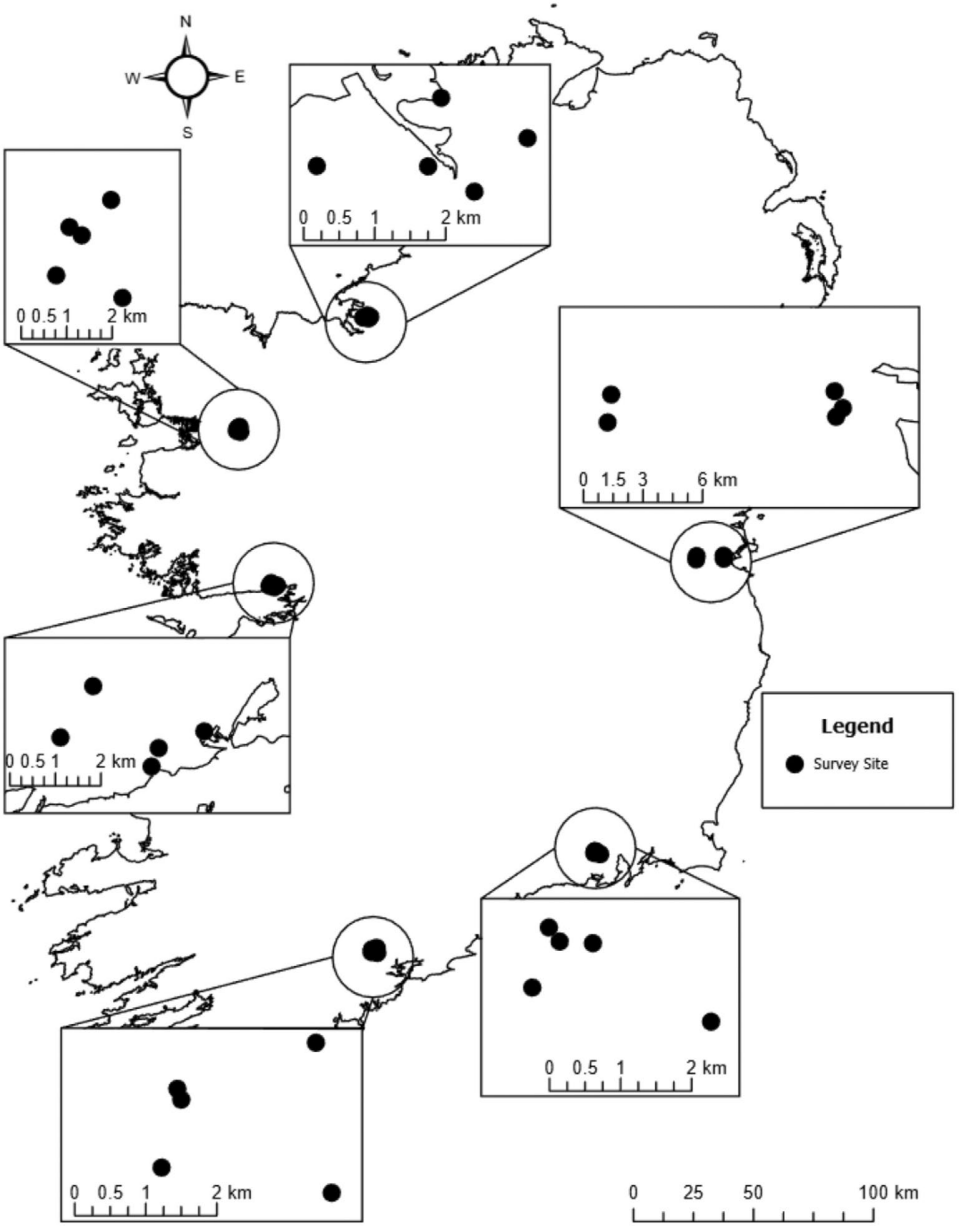
Synanthropic spider survey sites across the Republic of Ireland. Coordinates uploaded and mapped to the nearest 10 km grid using ArcGIS Pro v. 3.1.2 (ESRI 2023).

To test if there is a significant difference in synanthropic spider biodiversity between urban centres in Ireland, we implemented a nested surveying design. This was analysed using a mixed‐modelling framework with fixed (location, season) and random factors (survey site nested in each urban centre). This was implemented in surveying segments of fences across the country; one segment of fencing per survey site, five survey sites per urban centre (*n* = 6) (Figure 1). All surveys were conducted after sunset to ensure the most possible spider observations. After arriving at each site, the height of each fence surveyed was taken using measuring tape. Measuring tape was then used to portion a 10 m length of fencing, marking the survey's scope prior to each observation and allowing the surface area to be calculated, to infer the density of each spider community per m^2^. This allowed for the standardisation of fence sizing between sites, acting as a two‐dimensional “net” for analysis purposes. Observations of living spiders were recorded at each site over the course of one man‐hour. Additionally, observations of possible prey species, such as flying or crawling insects, were recorded (as present/absent) at each site within the same period. Surveys of sites in each urban centre were completed in 1 or 2 days, after sunset, depending on weather and accessibility. Individual sites were sampled four times over the course of 11 months (September 2022–July 2023), roughly corresponding with the four seasons.

To visually identify spiders in situ, the identification keys in the field guide *Britain's Spiders* (Bee et al. 2020) as well as the identification chart for *S. nobilis* in Dugon et al. (2017) were consulted. Species that could not be reliably identified to the species level by visual inspection alone were instead recorded at the genus level. Specimens that either were too small to show distinct characteristics (developmentally or otherwise) or escaped from view before they could be identified were recorded as “Unidentified.” Through this framework, we identified 20 spider species, belonging to 11 individual families, occupying fence microhabitats in Ireland (Table 1).

**TABLE 1:** Total abundance of synanthropic spiders surveyed in Ireland (September 2022–July 2023). Specimens were observed occupying fence/railing microhabitats and identified to the family, genus or species level using the field guide Britain's Spiders (Bee et al. 2020) and the *Steatoda nobilis* identification guide in Dugon et al. (2017).

Genus/Species	Family	Synanthropic status	Total	Relative abundance (%)
*Zygiella x‐notata*	Araneidae	Synanthropic	1835	59.62
*Steatoda nobilis*	Theridiidae	Synanthropic	1090	35.41
*Nuctenea umbratica*	Araneidae	Synanthropic	51	1.66
*Metellina* sp.	Tetragnathidae	Not typically synanthropic	22	0.71
*Segestria senoculata*	Segestriidae	Synanthropic	13	0.42
*Clubiona* sp.	Clubionidae	Not typically synanthropic	7	0.23
*Linyphiidae* sp.	Linyphiidae	N/A (varies by species)	5	0.16
*Amaurobius* sp. *(similis/fenestralis)*	Amaurobiidae	Synanthropic	5	0.16
*Pholcus phalangioides*	Pholcidae	Synanthropic	4	0.13
*Enoplognatha ovata*	Theridiidae	Not typically synanthropic	3	0.10
*Holocnemus pluchei*	Pholcidae	Synanthropic	3	0.10
*Steatoda grossa*	Theridiidae	Synanthropic	3	0.10
*Araneus diadematus*	Araneidae	Synanthropic	3	0.10
*Textrix denticulata*	Agelenidae	Synanthropic	2	0.07
*Pardosa* sp.	Lycosidae	N/A (varies by species)	2	0.07
*Larinioides* sp.	Araneidae	Synanthropic	1	0.03
*Meta menardi*	Tetragnathidae	Not typically synanthropic	1	0.03
*Scotophaeus blackwalli*	Gnaphosidae	Synanthropic	1	0.03
*Amaurobius ferox*	Amaurobiidae	Synanthropic	1	0.03
*Eratigena* sp.	Agelenidae	Synanthropic	1	0.03
Unidentified			25	0.81
Total			3078	100

## 3 Statistical Analysis

Rank‐abundance plots (also called Whittaker plots) were generated from the abundances quantified per m^2^, to compare the species richness and evenness of each urban centre, which was surveyed during each surveying cycle. These visualisations use the data collected from the spider biodiversity survey to represent both the “proportion of abundance” (how evenly distributed the species were observed at each location) against the species “rank” (how many species were observed at each location; ranked from most common to least common). The species assemblage was predominantly composed of one or two species, acting as extreme outliers, so a normal distribution could not be assumed. Because of this, non‐parametric testing was employed in the statistical analysis. Thus, the Whittaker plots were analysed for significant differences in the shapes of the curves using the Kolmogorov–Smirnov test. This non‐parametric procedure tests the null hypothesis that all the locations are equal in relative abundance at each surveying cycle.

To compare the alpha diversity of spider species at each urban centre surveyed, a Mann–Whitney *U* test was conducted to compare data between individual sites. This non‐parametric test is used to compare the medians of the two groups to assess whether they are significantly similar or different under the null hypothesis (Siegel 1957). The Mann–Whitney *U* test was used to determine whether there is a significant difference between synanthropic spider abundance and the presence or absence of possible prey items. The *U* test was performed on the Shannon diversity (*H*) and equitability (*E*_*H*_) indices of each individual survey repeat. These indices measure the biodiversity of each site by incorporating both species richness (*S*) and the proportion of species occupying each microhabitat (*p*_*i*_), in the case of the Shannon diversity index.

$$\begin{array}{c} H=-\sum_{i=1}^{S}p_{i}\ln\left(p_{i}\right)\\ E_{H}=\frac{H}{\ln\left(S\right)} \end{array}$$

To compare the synanthropic spider populations by locality, season, and prey availability, we utilised Bray–Curtis dissimilarity analysis (Bray and Curtis 1957). For analysis of location and seasonal differences, species densities (no./m^2^) were combined by urban centre per season. A Bray–Curtis dissimilarity matrix was then calculated as a measure of beta diversity using the ‘vegan’ package in R (Oksanen et al. 2025). Since the prey abundance data were per individual survey site, a separate Bray–Curtis dissimilarity matrix was calculated per individual survey. For each variable tested, a permutation‐based multivariate analysis of variance (PERMANOVA), which tests the differences in similarities between groups under the null hypothesis, was estimated. This was used to determine if the survey groups significantly differed by each variable. Non‐metric multidimensional scaling (NMDS) and UPGMA cluster analysis were then used to visualise differences in dispersion between the survey groups. To verify that results were not confounded by factors of dispersion, a test of homogeneity of dispersion (PERMDISP), with 999 permutations, was also performed using the ‘vegan’ package in R (Oksanen et al. 2025).

To examine how climate and seasonal weather patterns contribute to the assemblage of synanthropic spiders, publicly accessible weather data was collected from the national meteorological service, Met Éireann [https://www.met.ie], through its “Daily Data” database (Gleeson et al. 2017). The relevant variables logged for each sampling session were temperature (daily maximum and minimum) (°C), mean wind speed (knots), and rainfall (mm). Not every urban centre included in this study has a station within its geographical boundaries, and some others do not produce data for every variable (e.g., some stations do not produce mean wind speed data). As a result, stations were chosen on the basis of their proximity to the study area, and the next closest station was used if data were unavailable (Table S2). Meteorological data were then compared to the total Shannon diversity and equitability indices for each survey.

Apart from the linear regression metrics produced in Microsoft Excel, statistical analyses were performed using RStudio (RStudio 2020) which utilises R v.4.2.1 (2021).

## 4 Results

### 4.1 Population Demographics

#### 4.1.1 Relative Abundance of Spider Species in Urban Microhabitats

**FIGURE 2:**
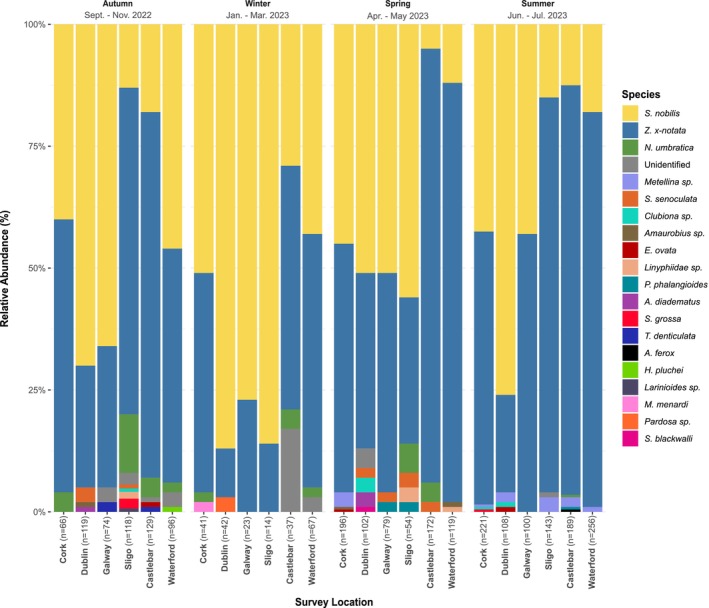
Relative abundance of spider species occupying urban microhabitats in Ireland (September 2022 to July 2023). Stacked bar charts showing the relative abundance of spider species identified in six urban centres in Ireland over four survey cycles.

In total, 3078 spiders belonging to 20 identifiable species were observed occupying fence microhabitats across all sampling sites (Table 1). Additionally, a total of 25 unidentified specimens were tallied over the four seasonal sampling cycles, representing less than 1% of the total spiders observed. Of the species identified, *S. nobilis* and *Z. x‐notata* dominate the assemblage (Figure 2); together comprising over 95% spider abundance over the survey period. We also found that *S. nobilis* and *Z. x‐notata* were absent from very few sites across the entirety of our study (*n* = 13 per species). Seven of the species were common synanthropic species such as *Zygiella x‐notata* , *S. nobilis* , *Nuctenea umbratica* , *Scotophaeus blackwalli* (Thorell, 1871), *Eratigena* sp. (Koch, 1843), and two species of cellar spiders, *Holocnemus pluchei* (Scopoli, 1763) and *Pholcus phalangioides* (Fuesslin, 1775). Also documented were four species that are not usually found in urban fence habitats. These include *Enoplognatha ovata* (Clerck, 1757), *Meta menardi* (Latreille, 1804), and several specimens from the genera *Clubiona* (Latreille, 1804) and *Metellina* (Chamberlin & Ivie, 1941).

#### 4.1.2 Species Richness and Evenness Between Urban Centres

**FIGURE 3:**
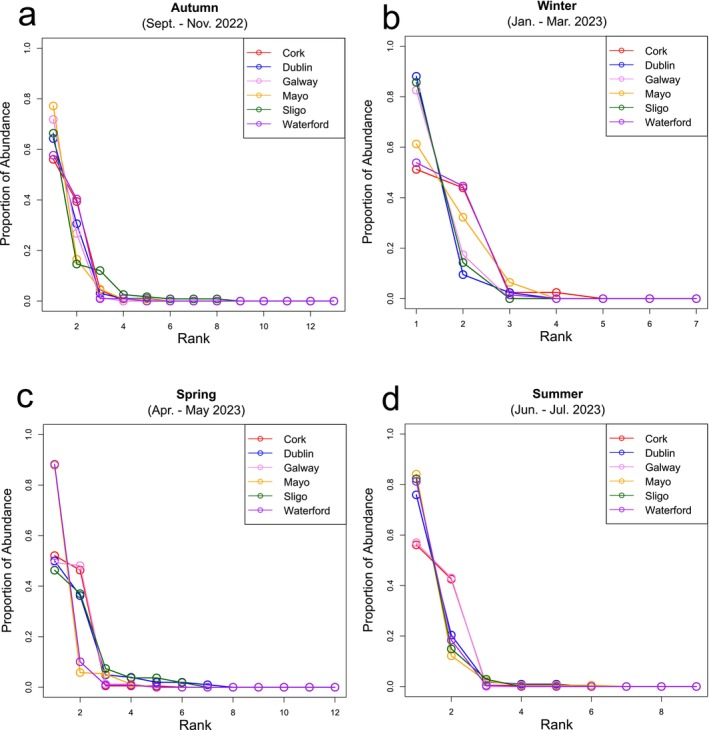
Species rank abundance charts representing Irish synanthropic spider species richness and evenness. The curves of six urban centres in Ireland (labelled here by county) are shown on a seasonal basis for (a) Autumn 2022, (b) Winter 2022/23, (c) Spring 2023, and (d) Summer 2023. Kolmogorov–Smirnov tests indicate no significant difference between any of the curves within their respective season (*p* < 0.05).

**FIGURE 4:**
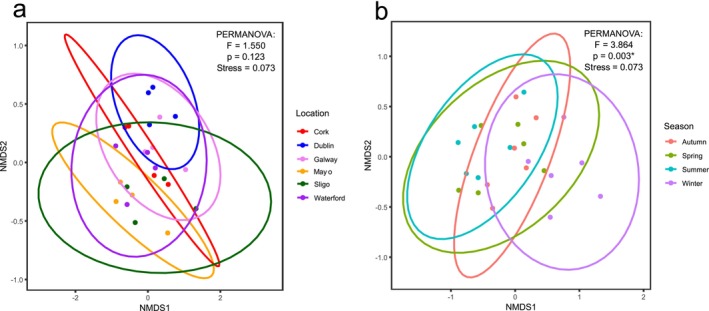
Non‐metric multidimensional scaling (NMDS) ordination of and PERMANOVA test of synanthropic spider assemblages on the basis of Bray–Curtis dissimilarity. Ellipses indicate groupings by (a) location (labelled here by county) and (b) season. PERMANOVA results are reported in the corner of each visualisation, * indicates a significance of *p* < 0.05.

**FIGURE 5:**
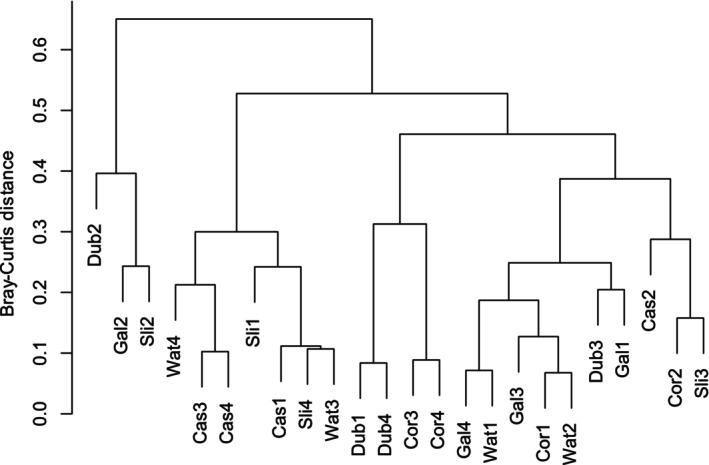
UPGMA cluster analysis using Bray–Curtis dissimilarity. Dendrogram showing the dissimilarity in beta diversity between six urban centres in Ireland (abbreviated here by location) over four survey cycles (1–4).

To compare the alpha diversity (i.e., species richness and evenness) of each urban centre over the surveying period, rank abundance charts were generated for each season and compared using the Kolmogorov–Smirnov Test at the selected significance level (*p* < 0.05) (Figure 3). Within each individual season, no significant differences between the vector series were detected, indicating no significant difference in species richness and evenness between the urban centres surveyed. Beta diversity analyses (i.e., comparing similarities between the survey sites) were performed using PERMANOVA tests at the same selected significance level (*p* < 0.05). These analyses indicated that the composition of the synanthropic spider populations did not significantly vary by location (Pseudo‐F = 1.550, *p* = 0.123) but did vary significantly by sampling season (Pseudo‐F = 3.864, *p* = 0.003) (Figure 4). Results of the PERMDISP test reveal no significant dispersion effect affecting the results of the model, confirming the significance of seasonality on the population. Additionally, UPGMA cluster analysis of the Bray–Curtis dissimilarity matrix revealed differences between the synanthropic spider populations in context with both location and season sampled (Figure 5).

### 4.2 Environmental and Ecological Factors

#### 4.2.1 Effect of Prey Presence/Absence on Synanthropic Spider Biodiversity

**FIGURE 6:**
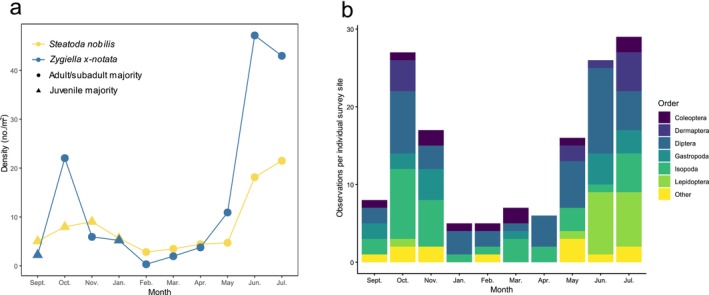
General phenology and prey diversity of *S. nobilis* and *Z. x‐notata* . The age class majority of the two most common spider species, (a) and observations of potential prey organisms, (b) surveyed per month over four survey cycles.

**FIGURE 7:**
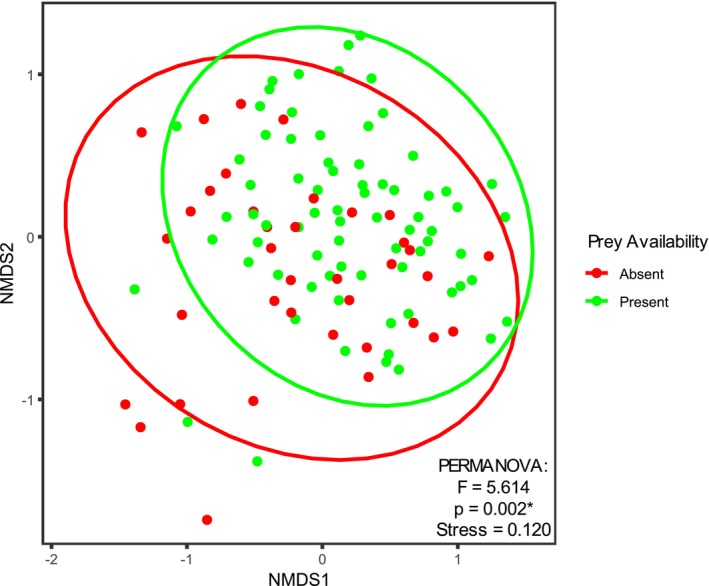
NMDS ordination of and PERMANOVA test of synanthropic spider assemblages per individual survey site on the basis of Bray–Curtis dissimilarity. Ellipses indicate groupings by prey presence or absence over survey duration. PERMANOVA results are reported in the corner of each visualisation, * indicates a significance of *p* < 0.05.

Basic identification of age class majority and potential prey items was conducted over the course of the study (Figure 6). Possible prey organisms recorded during the surveys included Dermapterans (earwigs), Dipterans (house flies, crane flies), Gastropods (snails, slugs), Isopods (woodlice), Lepidopterans (moths), and a wide range of Coleopterans (ladybirds, weevils, ground beetles). Mann–Whitney *U* testing revealed a significant difference (*U* = 1670.5, *p* = 0.023) in the Shannon diversity indices between sites that were observed as having possible prey items (presenting higher species richness) and those that were not (presenting lower species richness). However, there was no significant difference (*U* = 1577.5, *p* = 0.605) in the Shannon equitability indices between the sites that can be explained by the presence/absence of prey. A PERMANOVA test was performed to compare individual survey sites by prey availability. This analysis also indicated a significant difference between sites with and without potential prey items (Pseudo‐F = 5.614, *p* = 0.002) (Figure 7). PERMDISP testing on this model did not reveal any significant dispersion effect affecting the results of the PERMANOVA, confirming the significance of prey availability on the population.

#### 4.2.2 Effect of Bioclimatic Variables on Synanthropic Spider Biodiversity

**FIGURE 8:**
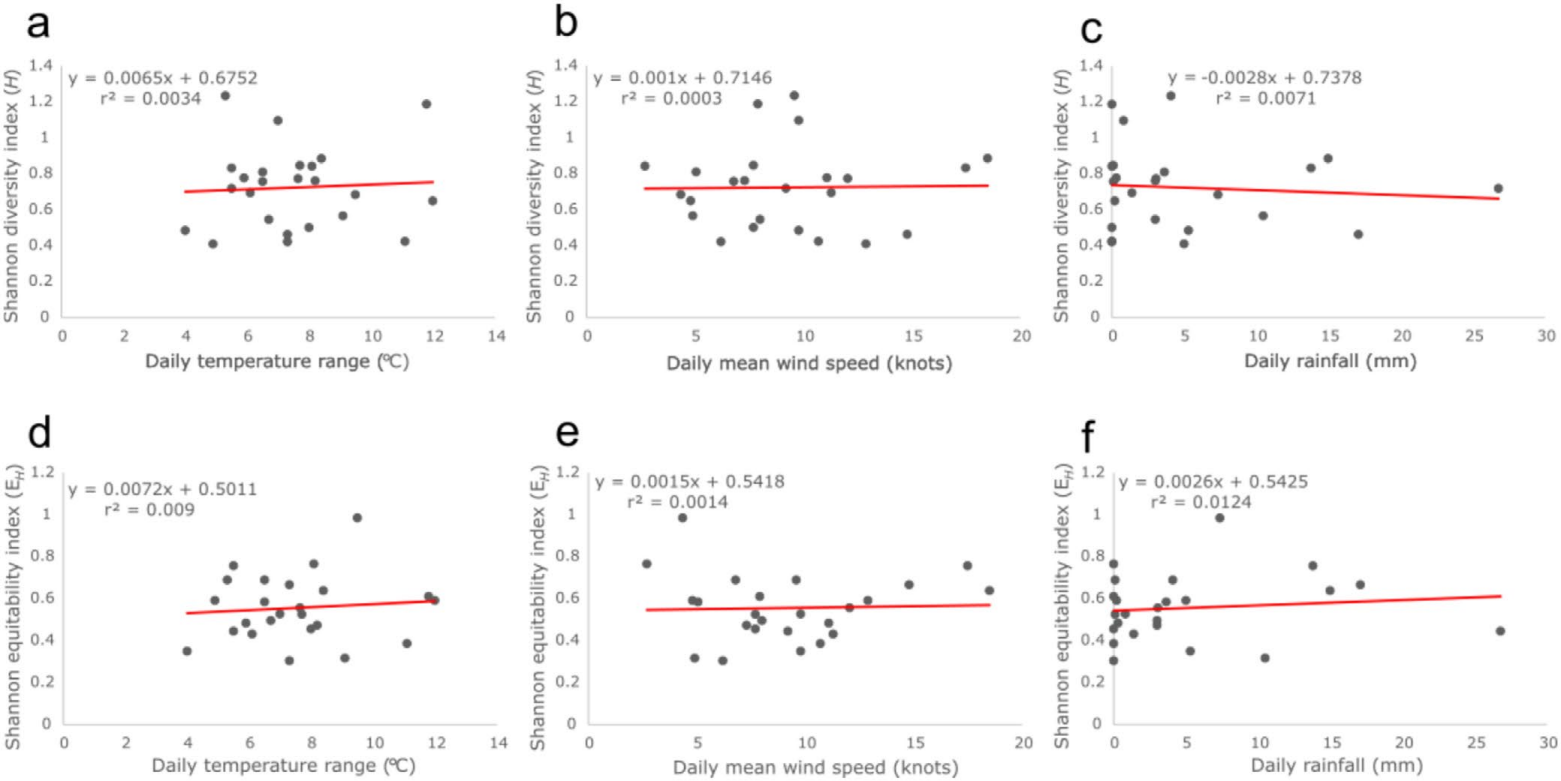
Scatterplots comparing daily climate data to the synanthropic spider biodiversity indices of urban centres in Ireland. The total Shannon diversity (a–c) and equitability indices (d–f) from six urban centres in Ireland (*n* = 24) were charted against three climate variables (temperature range (°C), mean wind speed (knots), rainfall (mm)). Bioclimatic variables for each site were gathered from the Met Éireann “Daily Data” database (Gleeson et al. 2017).

Linear regression indicates that none of the bioclimatic variables explain more than 1.24% of the species richness or evenness trends at each of the urban centres, according to their respective *r* ^2^ metrics (Figure 8). This could indicate that synanthropic spider population diversity, within favourable microhabitats in Ireland, is generally unaffected by variations in daily weather patterns.

## 5 Discussion

To analyse the reach of *S. nobilis* since its arrival in Ireland in the 1990s (Dugon et al. 2017; Nolan 1999), we surveyed spider assemblages in six major urban centres over an 11‐month period. Of the 20 discernible species, seven spider species discovered inhabiting these urban microhabitats (*Metellina* sp., *Clubiona* sp., *Linyphiidae* sp., *Enoplognatha ovata* , *Tetrix denticulata*, *Pardosa* sp., and *Meta menardi* ) are largely considered not to be synanthropic. Most of these spiders establish in garden or woodland areas, whereas some, such as *M. menardi* , typically occupy more extreme habitats (e.g., cave systems) (Bee et al. 2020). Considering that fences usually border gardens, churchyards, and parks in Ireland, it may be that they act as a crossover or junction for synanthropic and non‐synanthropic spiders alike. However, non‐synanthropic species observed make up no more than 0.5% of the total spider assemblage surveyed over the 11‐month period.

Of the synanthropic species documented within the 11‐month survey period, *S. nobilis* (35.41%) and *Z. x‐notata* (59.62%) contribute to the majority of the assemblage in favourable urban microhabitats across Ireland. Although both species were present in every urban centre throughout the study, *S. nobilis* and *Z. x‐notata* do present an expected degree of temporal variation, seen in the significant difference in beta diversity between the seasons surveyed. Both species were accidentally introduced to the island, but the high abundance of *S. nobilis* in synanthropic microhabitats nationwide is remarkable, since it was established in Ireland in the late 1990s, whereas *Z. x‐notata* was first documented in Ireland in the late 1800s (Dugon et al. 2017; Rayner et al. 2022; Workman 1880). We found no significant difference in both alpha and beta diversity between urban locations in Ireland. This could indicate a certain degree of biotic homogenisation in synanthropic spider populations in Ireland, potentially being influenced by the expansion of *S. nobilis* in urban habitats across the island.

Despite likely being the first large *S. nobilis* population centre in Ireland (Dugon et al. 2017), Dublin's St. Stephen's Green showed surprising levels of diversity across the study, with seven spider species being documented within the survey area in Spring 2023. Unlike other locations in Dublin, St. Stephen's Green is a densely wooded public park where pesticide use has been reduced. The recent conservation efforts in the park may explain the site's increased spider diversity compared to elsewhere in Dublin, creating a novel, urban refuge for Irish invertebrates. Likewise, the towns of Sligo and Castlebar, both located in the Northwest (i.e., the furthest from the hypothesised entry point of *S. nobilis* on the East coast) had high diversity with more non‐synanthropic species occupying urban microhabitats than the other urban centres surveyed. This may support the hypothesis that *S. nobilis* entered the island via the major port cities in the Centre‐East and dispersed gradually to the geographically more isolated Northwest.

Large populations of *S. nobilis* and *Z. x‐notata* are present throughout the year, which may indicate some level of coexistence between the species within urban environments. Although they likely compete for much of the same resources, *S. nobilis* and *Z. x‐notata* were observed building webs and hides mere centimetres away from one another. Although both *Z. x‐notata* and *S. nobilis* use similar hunting strategies (sit and wait), their different strategies of web building (orb‐webs for *Z. x‐notata* and tangle webs for *S. nobilis* ) may contribute to this proximity (Dugon et al. 2017; Dugon et al. 2023; Dunbar, Ennis, et al. 2018; Dunbar et al. 2022). Aggregative behaviour has been reported within populations of *Z. x‐notata* (Leborgne and Pasquet 1987), but interspecies and intraspecies aggregation of adult *S. nobilis* has yet to be documented. This reinforces the claim that the different ways that *S. nobilis* and *Z. x‐notata* utilise fences likely, in part, facilitate their coexistence despite competition for similar resources.

To explore how prey availability affects synanthropic spider biodiversity in Ireland, we gathered presence and absence data of possible prey items from each individual site repeated over 11 months. Considering that most spider species depend greatly on prey availability to fully complete their lifecycle (Bucher and Entling 2011; Foelix 1996), it was not surprising that there was a significant difference in alpha and beta diversity between fences with and without potential prey observed. However, since observations of prey availability are directly affected by the spiders themselves, it is not possible to discern a direct correlation using the data we collected over the course of the 11‐month sampling period. To properly gain insight into the complex impact that prey availability and diversity have on *S. nobilis* and other synanthropic spiders, and vice versa, diet and prey abundance studies could be done in situ. The same can be said about the phenology of synanthropic spiders, as their age demographics over a study period can be used to gain another dimension on their relationship with biotic and abiotic factors (Barnes et al. 2023). Concurrently, we found that species evenness (*E*_*H*_) was not significantly affected by the presence or absence of prey. Since species evenness is similarly uneven in favourable fence microhabitats, it can be inferred that both *S. nobilis* and *Z. x‐notata* are potentially the most abundant species in similar urban microhabitats across the island.

Regarding climatic variations as contributing factors to synanthropic spider biodiversity in Ireland, we found that daily weather conditions (day‐night temperatures, rainfall, and wind speeds) do not have a significant effect on synanthropic spider species richness or evenness in Ireland. As Ireland is prone to relatively cool, wet, and windy winters, it is likely that any species that has an established population on the island would require mechanisms to survive the local climatic conditions. However, these mechanisms are not as necessary within urban environments, which provide increased opportunity for protection as well as higher mean temperatures that create gradients that can accommodate a variety of arthropod heat tolerances (Frank and Backe 2023). Regardless, *S. nobilis* seems to require shelter to overwinter, including a den or retreat. On fences, appropriate retreats for spiders, including holes or nooks in their wooden or cast‐iron structures, are relatively limited. This introduces a unique situation where intraguild competition may be at play between *S. nobilis* and other spiders attempting to gain control of a retreat. Since synanthropic spider diversity in these microhabitats is not significantly affected by abiotic factors, other factors may be influencing the composition of the assemblage. Given that *S. nobilis* is likely to outcompete Irish spiders (Rayner et al. 2022), this yearly struggle may have some impact on other spider populations occupying favourable microhabitats in urban areas. Further comparative studies with certain synanthropic spider populations not containing *S. nobilis* and *Z. x‐notata* could reveal if their dominance is driven by direct displacement of non‐synanthropic species or superior adaptation to biotic and abiotic conditions in urban environments.

The Noble false widow spider, *Steatoda nobilis* , has become well established on the island of Ireland. Although *Z. x‐notata* has been able to coexist with *S. nobilis* on fence microhabitats, their abundance may affect the ability of other synanthropic spider species to utilise such favourable microhabitats. As global urbanisation continues to progress, cosmopolitan predators such as *S. nobilis* may have a distinct advantage in the arena of intraguild competition in urban ecosystems. Its abundance and success in colonising the island of Ireland over the last 25 years suggest that *S. nobilis* may be acting negatively on native spider species and may require acknowledgement and immediate action associated with an “invasive alien species” classification under the UN Convention on Biological Diversity's guidelines (Global Biodiversity Outlook 5 2020).

## Author Contributions

**Brandon L. Collier:** conceptualization (equal), data curation (equal), formal analysis (equal), funding acquisition (equal), investigation (equal), methodology (equal), project administration (equal), software (equal), validation (equal), visualization (equal), writing – original draft (equal). **Dayle Leonard:** conceptualization (equal), investigation (equal), methodology (equal), writing – review and editing (equal). **Keith Lyons:** conceptualization (equal), investigation (equal), methodology (equal), writing – review and editing (equal). **John P. Dunbar:** conceptualization (equal), investigation (equal), methodology (equal), writing – review and editing (equal). **Colin Lawton:** conceptualization (equal), conceptualization (equal), formal analysis (equal), formal analysis (equal), methodology (equal), methodology (equal), supervision (equal), supervision (equal), writing – review and editing (equal), writing – review and editing (equal). **Michel M. Dugon:** conceptualization (equal), formal analysis (equal), funding acquisition (equal), methodology (equal), project administration (equal), resources (equal), writing – review and editing (equal).

## Funding

This work was financed by the University of Galway Hardiman Research Scholarship held by Brandon L. Collier.

## Disclosure

All authors contributed to the conception and design of the study. Data collection and preparation were conducted by Brandon L. Collier, Dayle Leonard, Keith Lyons, and John P. Dunbar. Data analysis was performed by Brandon L. Collier with guidance from Michel M. Dugon and Colin Lawton. The first draft of the manuscript was written by Brandon L. Collier, and all authors provided comments and revisions to previous versions of the manuscript. All authors read and approved the final manuscript.

## Conflicts of Interest

The authors declare no conflicts of interest.

## Supporting information


**Table S1:** Microhabitats in Ireland surveyed for synanthropic spider biodiversity. Sites were randomly chosen from a generated list of possible survey sites by the number generator RANDOM.ORG (Haahr 2023). Coordinates were obtained using Google Earth Pro (Map of the Republic of Ireland 2022).
**Table S2:**: Met Éireann weather stations used to collect daily climate data. Data were collected from https://www.met.ie/climate/available‐data/daily‐data (Gleeson et al. 2017).

**Data S1:** ece373193‐sup‐0002‐supinfo02.xlsx.

## Data Availability

Supplementary figures are placed in the Appendix, and all the required data are uploaded as Supporting Information.
