# Supplementary material for: Survey of Synanthropic Spiders in Ireland Reveals Expansion and Dominance of the Invasive Noble False Widow Steatoda nobilis in Urban Habitats (Araneae: Theridiidae)
Source: Ecol Evol. 2026 Mar 10;16(3):e73193. doi: 10.1002/ece3.73193 (PMC12975292; doi:10.1002/ece3.73193)
Supplement: Supplementary file 1 — Table S1: Microhabitats in Ireland surveyed for synanthropic spider biodiversity. Sites were randomly chosen from a generated list of possible survey sites by the number generator RANDOM.ORG (Haahr 2023). Coordinates were obtained using Google Earth Pro (Map of the Republic of Ireland 2022). Table S2:: Met Éireann weather stations used to collect daily climate data. Data were collected from https://www.met.ie/climate/available‐data/daily‐data (Gleeson et al. 2017). [file ECE3-16-e73193-s001.docx]

APPENDIX

**Supplementary Table 1: Microhabitats in Ireland surveyed for synanthropic spider biodiversity.** Sites were randomly chosen from a generated list of possible survey sites by the number generator RANDOM.ORG (Haahr, 2023). Coordinates were obtained using Google Earth Pro (*Map of the Republic of Ireland*, 2022).

| ***Location*** | ***Location Type*** | ***Coordinates*** |
| --- | --- | --- |
| Co. Cork – Belfield Bus Stop | Housing estate | 51.89125, -8.445549 |
| Co. Cork – Bob and Joan's Walk | Cathedral | 51.903201, -8.476178 |
| Co. Cork – Cathedral of St. Mary and St. Anne | Cathedral | 51.904582, -8.476959 |
| Co. Cork – St. Fin Barre's Cathedral | Cathedral | 51.894616, -8.480297 |
| Co. Cork – St. Joseph's Church | Cathedral | 51.910294, -8.448551 |
| Co. Dublin – Balgaddy Road Bus Stop | Housing estate | 53.340421, -6.430734 |
| Co. Dublin – St. Mary's Pro Cathedral | Cathedral | 53.350711, -6.258406 |
| Co. Dublin – St. Steven's Green North Bus Stop | Public park | 53.339175, -6.258035 |
| Co. Dublin – Trinity College Dublin Park | Public park | 53.342997, -6.252675 |
| Co. Dublin – Willsbrook Park Bus Stop | Public park | 53.352979, -6.427248 |
| Co. Galway – Altan Apartments | Apartment complex | 53.268997, -9.101294 |
| Co. Galway – Churchfields House | Art studio | 53.266922, -9.068894 |
| Co. Galway – Portmore Apartments | Apartment complex | 53.270225, -9.053856 |
| Co. Galway – Radharc An Chlair | Apartment complex | 53.263269, -9.071244 |
| Co. Galway – Seafield Apartments | Housing estate | 53.279172, -9.090533 |
| Co. Mayo – Church of the Holy Rosary | Cathedral | 53.858292, -9.30084 |
| Co. Mayo – Glen Fort | Housing estate | 53.859889, -9.304946 |
| Co. Mayo – Hawthorn Village | Apartment complex | 53.845942, -9.287117 |
| Co. Mayo – Lough Lannagh Playground | Playground | 53.85034, -9.309295 |
| Co. Mayo – Turlough Road | Housing estate | 53.865316, -9.291075 |
| Co. Sligo – Abbey Street | Cathedral | 54.270644, -8.470229 |
| Co. Sligo – Cartron Village Bus Stop | Public park | 54.282564, -8.477263 |
| Co. Sligo – First Sea Road Bus Stop | Apartment complex | 54.274056, -8.504245 |
| Co. Sligo – Gateway Apartments | Apartment complex | 54.277377, -8.458674 |
| Co. Sligo – The Old Mill | Housing estate | 54.2739, -8.480198 |
| Co. Waterford – Alexander Street | Housing estate | 52.259485, -7.112954 |
| Co. Waterford – Manor Village Apartments | Apartment complex | 52.25193, -7.116713 |
| Co. Waterford – Maritana Gate | Apartment complex | 52.257349, -7.103977 |
| Co. Waterford – St. John's Church | Cathedral | 52.25767, -7.110817 |
| Co. Waterford – University Hospital Waterford | Public park | 52.247008, -7.080055 |

**Supplementary Table 2: Met Éireann weather stations used to collect daily climate data.** Data was collected from https://www.met.ie/climate/available-data/daily-data (Gleeson et al., 2017).

| ***Location*** | ***Station Location (Temperature)*** | ***Station Location (Wind Speed)*** |
| --- | --- | --- |
| Co. Cork | Cork Airport | Cork Airport |
| Co. Dublin | Phoenix Park | Dublin Airport |
| Co. Galway | Athenry | Athenry |
| Co. Mayo | Claremorris | Claremorris |
| Co. Sligo | Markree Castle | Knock Airport |
| Co. Waterford | Johnstown Castle | Johnstown Castle |
